# Unpacking the interplay between psychological capital and wellbeing in entrepreneurs: the mediating role of burnout through conservation of resources theory

**DOI:** 10.3389/fpsyg.2025.1590554

**Published:** 2025-07-07

**Authors:** Sarfraz Ali Malak, Amal Abdulmajeed Qassim

**Affiliations:** ^1^Department of Business Administration, University of Sindh (Campus Dadu), Sindh, Pakistan; ^2^Department of Management, Faculty of Business Administration, University of Tabuk, Tabuk, Saudi Arabia

**Keywords:** psychological capital, psychological wellbeing, entrepreneurs, burnout, conservation of resources (COR) theory

## Abstract

**Introduction:**

Despite growing research on exploring positive psychological aspects of entrepreneurs’ well-being, insufficient studies exist in the context of entrepreneurs in Sindh, Pakistan. The role of burnout as a mediator in the relationship between PsyCap and psychological well-being (PWB), from the perspective of the Conservation of Resources (COR) theory, is underexplored.

**Methods:**

This study has adopted quantitative methods and a survey technique was used to gather cross-sectional data from entrepreneurs of Sindh Province, Pakistan. A questionnaire from past relevant studies has been used to collect responses from a sample of 309 respondents through a simple random sampling technique. The data were analyzed through Partial Least Square Structural Equation Modeling (PLS-SEM) in Smart-PLS version 4.

**Results:**

The results show that PsyCap correlates positively to PWB and negatively to burnout, in a significant way. In addition, findings also reveal that burnout mediates the interplay between PsyCap and PWB.

**Discussion:**

These research outcomes suggest that increased burnout results in lower positive psychological aspects that lead to the lower psychological well-being of entrepreneurs; however, increased PsyCap (hope, optimism, resilience and self-efficacy) buffers the burnout and maintains the entrepreneurs’ flourishing and healthy psychological well-being. Hence, the present study outcomes empirically validate COR theory in the context of entrepreneurship.

## Introduction

Entrepreneurs are key contributors to economic growth, innovation and employment generation; they are engaged in starting and running businesses (GEM, 2022). Entrepreneurs’ role becomes more challenging and vital in developing regions including Sindh Province, Pakistan due to economic uncertainty, weak infrastructure and lower entrepreneurial support. Besides, entrepreneurs face stressors, such as work pressures and overload, targets, aloneness, conflicts, and role ambiguity (Tahar, 2012). In Pakistan, due to challenges and insufficient support, the stressors cause burnout which negatively impacts the wellbeing and health of entrepreneurs. Losing wellbeing due to burnout impacts entrepreneurs’ businesses and hardly hits the entrepreneur’s positive functioning and fulfilling psychological needs. However, with the capacity of positive psychological aspects such as PsyCap (hope, optimism, resilience and self-efficacy) entrepreneurs can achieve good health and wellbeing and mitigate the burnout and stress effects through coping mechanisms. Literature insights stated that burnout impacts entrepreneurs personally by causing doubtfulness, anxiety and worsening health, and organizationally by decreasing productivity and profits, business loss and failure, higher absenteeism and business quitting behavior (Lechat and Torrès, 2016; Lewin and Sager, 2007; Wincent et al., 2008). Research evidence shows that burnout negatively influences the performance of businesses (Fatoki, 2019). Nascent entrepreneurs experience a severe impact of burnout on their ventures and health (Omrane et al., 2018). Due to the worst effects of stress and burnout on individuals and businesses, efforts were underway to explore the positive aspects of individuals. Finally, Seligman pioneered positive psychology; which emphasizes positive human functioning with positive abilities and strengths of individuals (Seligman and Csikszentmihalyi, 2000). According to these authors, the human positive aspects include wellbeing, happiness, positive traits, and the potential abilities that contribute to flourishing people and communities. Thus, empirical studies focused on exploring entrepreneurs from a positive psychological perspective, such as the interest in investigating entrepreneurs’ wellbeing and mental health (Stephan, 2018). Engaging in entrepreneurship is related to gratifying psychological needs which facilitates achieving subjective wellbeing (Nikolaev et al., 2020). PsyCap has been identified as a powerful resource for the positive functioning of individuals. Fred Luthans established the Psychological Capital (PsyCap) construct which is referred to as “an individual’s positive psychological state of development” (Luthans et al., 2007a,b). PsyCap consists of four components “Hope, (Self-) efficacy, Resilience, and Optimism” (Luthans and Youssef-Morgan, 2017). Empirical evidence revealed that PsyCap was positively correlated with entrepreneurs’ PWB (Baluku et al., 2018; Hmieleski and Carr, 2007). Entrepreneurs with higher levels of PsyCap assets became more successful in entrepreneurial activities (Avey et al., 2010; Juhdi et al., 2015; Luthans et al., 2007a,b; Paul and Devi, 2018). It is also reported that healthier PsyCap resources reduce the stress and burnout effects of entrepreneurs (Baron et al., 2016; Hmieleski and Carr, 2007). Ryff (1989) introduced the “psychological wellbeing (PWB)” construct having six elements such as environmental mastery, personal growth, positive relations with others, purpose in life, and self-acceptance. “Entrepreneurial wellbeing is a positive and distinctive mental state, which reflects entrepreneurs’ affective and cognitive experiences of engagement in entrepreneurship as the process of venture creation. These experiences are characterized by positive judgments of the entrepreneurial life and good feelings about it” (Shir, 2015, p. 76). Authors’ studies witnessed that wellbeing is the valuable outcome of entrepreneurs and it was observed that “psychological and coping” mechanism impacted on the entrepreneurs’ mental health (Shepherd et al., 2010; Uy et al., 2013). Involving in the process of entrepreneurship motivates entrepreneurs to fulfil basic psychological needs and achieve better PWB (Shepherd and Patzelt, 2017; Williams and Shepherd, 2016).

Burnout is caused by acute stress (Salami, 2011). Burnout is the result of being unsuccessful in achieving the goals and targets of a business or job which generates feelings of anxiety, fear and frustration (Freudenberger, 1974). Burnout shows the exhausting levels of individuals’ emotional, mental and physical aspects (Pines and Aronson, 1988). It can harm physically, mentally and financially to individuals and organizations as well (Cordes and Dougherty, 1993; Leiter et al., 2014; Maslach and Goldberg, 1998; Maslach and Leiter, 1997). In Pakistan, more stressors such as financial uncertainty, infrastructure challenges and low entrepreneurial support have negatively impacted entrepreneurial activities. Subsequently, Pakistan has a low entrepreneurship rate (GEI, 2019; GEM, 2012), thus, economic problems persist. Further, it can be argued that entrepreneurs have to perform strenuous work (Sheehan and St-Jean, 2014). This could lead to gratification or stress (Cadet and Chasseigne, 2012). The stress and burnout could be other reasons for a lower rate of entrepreneurship in Sindh and Pakistan as individuals face various stressors of economic and environmental challenges. Subsequently, it seems that it is difficult for individuals in Pakistan to engage in entrepreneurship and satisfy psychological-related needs, as more effort and skills are required to perform highly demanding entrepreneurial work but fewer abilities and potentials have deteriorating effects on them (Naik, 2012). People avoiding availing new business opportunities in Pakistan can be linked to the evidence of a risk aversion attitude (Hofstede, 2001). Therefore, building higher PsyCap resources and achieving good health and PWB is significant for entrepreneurs. As a result, the worst effects of burnout will lessen and entrepreneurs through resilience and coping will handle risks and uncertainties that lead to their success and thriving lives. Subsequently, entrepreneurship will boost and the economy will grow at full speed. A review of earlier research studies reveals that less empirical evidence exists on exploring the role of PsyCap in PWB, and the mediating role of burnout seems underexplored in the context of entrepreneurs of Sindh-Pakistan. The theoretical gap in applying COR theory remains unfilled, specifically in understanding the mechanism of how entrepreneurs manage and maintain psychological resources under stressors. Therefore, this study has been designed in the context of the entrepreneurs of Sindh (Pakistan); with the core purpose of examining the interplay between PsyCap and PWB. It also aims to investigate the mediating effect of burnout on the link between PsyCap in the perspective of COR theory. This study answers research questions; is PsyCap correlated to the PWB of entrepreneurs? Does PsyCap correlate to burnout? Does burnout mediate the relationship between PsyCap and PWB?

### Research gap

The prior empirical studies have focused more on health workers, such as examining the impact of PsyCap on burnout (Peng et al., 2025; Chen et al., 2024; Zambrano-Chumo and Guevara, 2024), and investigating the relationship between PsyCap and PWB (Munyod and Wattananonsakul, 2025; Nasria and Gara Bach Ouerdian, 2023). Since entrepreneurs play an important role in the economy. Entrepreneurs do not only provide valuable products and services by utilizing resources but their work is full of risk and uncertainty (Hisrich et al., 2010). Uncertain and risky situations create stress and burnout for entrepreneurs, which impacts their businesses and lives. Less literature is available on empirical investigations pertinent to entrepreneurs’ PsyCap, PWB and burnout, which provides a gap for this study. Entrepreneurs’ ability to assume a risk depends on the nations’ “entrepreneurial ecosystem” support (GEM, 2022). A study reported that people in Pakistan avoid taking risks (Hofstede, 2001). This risk aversion attitude causes no new business opportunities, hence, the rate of entrepreneurship remains low in Pakistan (GEI, 2019; GEM, 2012). Pakistan needs to devise policies to engage youth in the development of the economy (Hameed Khan, 2016). However, few steps have been taken to grow entrepreneurship among youth and to provide new business opportunities to better use their knowledge and skills in the economic growth of Pakistan (Aslam and Hasnu, 2016; Mahmood et al., 2017). Nevertheless, entrepreneurial activities are still low and the country’s economy grapples with various challenges. This enhances the research interest in entrepreneurship. While literature mounds in empirical evidence on entrepreneurs in Pakistan in context of mental health (Saraf, 2019), anxiety and depression (Hussain and Li, 2022), “attitude,” “perceived behavior control” “entrepreneurial intentions,” and “entrepreneurial motivation” (Alam, 2019), psychological capital resources (Sarwar et al., 2021), psychological factors (such as locus of control, need for independence, risk-taking, and emotional intelligence) were investigated (Qudus et al., 2022), locus of control (PsyCap) and wellbeing (Soomro et al., 2018), entrepreneurship and stress (Arshi et al., 2021). Nonetheless, the literature is deficient in providing practical evidence on the correlations among PsyCap, psychological wellbeing and burnout in the context of entrepreneurs of Sindh- Pakistan. There is also a need to fill a research gap in investigating burnout as a mediator in the relationship of PsyCap and PWB of entrepreneurs. The literature has insufficient evidence in the context of Sindh-Pakistan.

### Problem statement

Entrepreneurs’ tasks involve exploiting new business opportunities, taking decisions and handling risks and uncertainties which emerge from unexpected situations and challenges. In this regard, PsyCap is a significant resource for entrepreneurs (Newman et al., 2014). Entrepreneurs gratify their psychological desires by engaging in entrepreneurial activities which lead to wellbeing and better psychological functioning (Williams and Shepherd, 2016). More risks and challenges cause stress and burnout to entrepreneurs. Thus, PsyCap resources (hope, optimism, resilience and self-efficacy) are mandatory for entrepreneurs. Although, various studies exploring entrepreneurship in the context of Pakistan have been done, yet, there is a research gap; how does PsyCap correlate to PWB? How is burnout related to PWB? And how does burnout mediate the link between PsyCap and PWB? These research questions and relationships among variables seem underexplored and less focused in the context of entrepreneurs of Sindh (Pakistan). Empirical evidence on these themes could develop our understanding of the role of positive psychological aspects in achieving entrepreneurial goals, wellbeing and how it relates to burnout. These insights will contribute to stakeholders designing policies in Sindh, Pakistan to promote entrepreneurship and achieve economic growth.

## Literature review

The literature has been critically and carefully reviewed for the current study. This includes a discussion of the conservation of resources (COR) theory and logical debate on past studies related to PsyCap, PWB and burnout in the entrepreneurship context. Accordingly, the research gap, conceptual framework and hypotheses were developed based on this literature review.

### Theoretical base: conservation of resources theory

Hobfoll (1988, 1989) “conservation of resources (COR) theory” argues about the process whereby due to stressors individuals face situations of resource loss and gain. This theoretical base provides a framework for conducting investigations related to “traumatic stress,” and “burnout” as well as on “occupational burnout” (Hobfoll et al., 2018; Gorgievski and Hobfoll, 2008; Yin et al., 2020). These theory-supported insights contribute to understanding individuals’ abilities in handling burnout and stressful situations through coping and resilience. Therefore, The COR model discusses that people continuously work on developing, growing and safeguarding vital psychological assets required for their better functioning (Hobfoll, 1989; Hobfoll et al., 2018). In context to this study variables; PsyCap, Psychological wellbeing and burnout, the COR theory states that people put efforts in acquiring, retaining, and preserving their key personal, social and material resources. The personal resources include positive psychological potentials such as PsyCap (hope, optimism, resilience and efficacy). Social resources encompass family patronage, professional networking, community trust and mentorship. Material assets include financial resources (capital, funds, and profits), infrastructure, equipment and business support. COR theory further argues that feelings of stress are caused when these significant assets are under threat, lost or replenished insufficiently. In reaction to these stressors, individuals feel burnout which causes them to deplete their resources, referred to as resource loss (Hobfoll et al., 2018). This loss of resources badly affects the mental health and PWB of individuals. However, through a high level of PsyCap resources, the burnout effect is reduced and resources are refilled, which is referred to as resource gain (Hobfoll et al., 2018). Subsequently, improved health and better PWB are achieved. By applying COR theory to our research, it is argued that entrepreneurship is quite challenging, specifically in developing regions like Sindh (Pakistan). Entrepreneurs have to handle unpredictable situations with limited abilities, which creates stress and burnout. Increasing burnout damages and reduces the entrepreneurs’ psychological resources which worsens PWB. Under COR theory, each dimension of PsyCap such as hope, optimism, resilience and self-efficacy qualifies as a resource which assists entrepreneurs in addressing challenges, coping with stress, recovering from setbacks and performing tasks effectively. Hope as a resource allows entrepreneurs to find different pathways to achieve goals in case of hurdles. Optimism is a cognitive resource that helps entrepreneurs to have a positive outlook about succeeding in future. Resilience facilitates resource conservation supports entrepreneurs in bouncing back from worst situations and protects resources from loss. Self-efficacy works for entrepreneurs as resource acquisition and develops the belief of working effectively in achieving goals. When entrepreneurs face stressors, this causes a reduction of resources which leads to mental pain (DeNeve, 1999), and triggers withdrawing behavior which requires that PsyCap assets be refilled to meet the deficiency of means needed for coping (Fatima et al., 2018). Therefore, individuals improve their strengths and mental abilities and keep a good reservoir of PsyCap for better use in handling stressful conditions and meeting future challenges (Hobfoll et al., 2018). In addition, PsyCap resources such as hope, optimism, resilience and efficacy ameliorate positive psychological functioning, which is mandatory for entrepreneurs to fulfil psychological needs (Shir et al., 2019; Baron et al., 2016). This would lead entrepreneurs to achieve fulfilling, meaningful and thriving lives.

### Review of relevant prior research evidence

#### Psychological capital (PsyCap) and psychological wellbeing

PsyCap is a very important construct, consisting of hope, optimism, resilience and self-efficacy (Luthans et al., 2010). The PsyCap is more significant to entrepreneurs, as their jobs require handling various challenges and risks, achieving future goals and remaining resilient against adversities. The authors’ study evidenced that PsyCap was positively correlated with the PWB of caregivers of stroke patients (Munyod and Wattananonsakul, 2025). The research contribution of Khan et al. (2024) highlighted that augmenting PsyCap has contributed to enhancing students’ PWB. Similarly, it was observed in research results that PsyCap improved the workplace wellbeing of healthcare professionals (Nasria and Gara Bach Ouerdian, 2023). Research findings witnessed that better “psychological capital” increased the wellbeing of workers (Al Kahtani and M, 2022). In another study, it was reported that PsyCap impacted in a positive and significant way on the entrepreneurs’ work-related satisfaction, performance, attitude and citizenship actions in organizational settings, however, a negative effect was seen on hostile behavior (Wang et al., 2018). An empirical study by Baluku et al. (2018) witnessed that PsyCap related positively to the wellbeing of entrepreneurs. The authors’ investigation concluded that PsyCap correlated positively to psychological wellbeing (PWB) in principals of high schools (Malekitabar et al., 2017). Study argued that to boost more PWB of staff directly engaged in providing autism services, the healthier PsyCap resources need to be enhanced (Manzano-García and Ayala, 2017). PsyCap related positively to “hedonic” and “eudaimonic” wellbeing (Culbertson et al., 2010). The outcomes of the author’s work also revealed that the factors of PsyCap had a positive relationship with PWB in occupational health (Semmer et al., 2008; Hansen et al., 2015). The literature review also states that PsyCap is postively related to job performance and job satisfaction in the organizations (Luthans and Youssef, 2007). Research insights of Stajkovic (2006) presented that to make employees feel and work better, it is mandatory to enhance positive psychological aspects of staff. The investigation observed that PsyCap decreased the bad consequences of work stressors that hamper job satisfaction among workers (Hmieleski and Carr, 2007). Literature witnesses the great importance of PsyCap for job satisfaction, employee PWB, employee performance and betterment of organizations too. Therefore, the PsyCap construct introduced by Luthans et al. (2007a,b) needs to be explored and understood from the perspective of entrepreneurship. PsyCap is famous for its four factors “hope,” “(self)-efficacy,” “resilience,” and “optimism,” and is referred to as “HERO.” Hope is referred to as the person’s way of thinking and attitude (Snyder, 1994), this is required for performing any work. Self-efficacy represents the belief of the human in his abilities (Luthans et al., 2010). Optimism denotes positive expectations of the people (Luthans et al., 2010). Resilience shows an individual’s capacity to return from the worst conditions (Masten and Reed, 2002). These abilities are also required for entrepreneurs as they have to work hard, achieve goals, assume risks and challenges and address adverse situations.

The psychological Wellbeing (PWB) construct proposed by Ryff comprises six dimensions “self-acceptance,” “positive relations,” “autonomy,” “environmental mastery,” “purpose in life,” and “personal growth” (Ryff, 1989, 2014, 2019; Ryff and Keyes, 1995). These components of PWB refer to an individual’s collective qualities of keeping positive thinking for oneself, developing trustable relations with others, having confidence in making own decisions and judgments, handling environmental challenges, and having purposeful life and personal successes. Similarly, entrepreneurs face risks and uncertainties in starting and running businesses where they make decisions, resolve conflicts, build ties with other stakeholders, respond to environmental challenges and realize personal growth. PWB is highly required by entrepreneurs (Shir et al., 2019; Uy et al., 2013), it facilitates achieving goals, responding to outside threats with personal strengths, and deciding and maintaining networking for greater business and personal growth. How PsyCap correlate PWB? Need to be tested. Thus, it is hypothesized here that;

*H1*: PsyCap is positively correlated to the PWB of the entrepreneurs

In the perspective of COR theory, the entrepreneurs with higher levels of PsyCap assets have greater mental resources which help them in coping stress and subsequently enhance PWB.

### Psychological capital and entrepreneurial burnout

Burnout is caused by severe stress resulting from failures, challenges, risks, uncertainties and problems that individuals face. Evidence exists on the relationship between PsyCap and burnout in diverse workplace settings, such as the study of Peng et al. (2025) reported that a group of Nurses with higher levels of PsyCap caused lower levels of emotional exhaustion. Likewise, the investigation revealed that PsyCap lowers burnout among healthcare professionals (Zambrano-Chumo and Guevara, 2024). Research contributions of Chen et al. (2024) showed that PsyCap negatively related to the burnout among primary healthcare workers. Nurses with increased levels of PsyCap reported experiencing less burnout (Xue et al., 2024). Empirical studies undertaken on PsyCap and burnout relationship in the context of entrepreneurs, the insights reported that PsyCap negatively impacted the stress of entrepreneurs, which means entrepreneurs having good mental resources helped them to reduce stress and anxiety levels (Feng and Chen, 2020; Baron et al., 2016). PsyCap resources mitigated workplace burnout in entrepreneurs (Hmieleski and Carr, 2007). Some author’s work also witnessed that employees’ healthier PsyCap assets (such as “self-efficacy,” “hope,” “optimism,” and “resilience”) helped them to decrease bad consequences of burnout and increased wellbeing (Manzano-García and Ayala, 2017). Results of the study by Malekitabar et al. (2017) presented that PsyCap resources enhance PWB and reduce burnout effects. It was also revealed by the research findings that PsyCap develops potential abilities of staff which ameliorates burnout effects by managing adversities and challenges and also creates healthier PWB (Manzano-Garcia and Ayala-Calvo, 2013). Similarly, other investigations showed that PsyCap strengthens PWB and relaxes the burnout effects of the staff (Avey et al., 2010). Authors discussed that Burnout refers to the individual’s “physical,” “emotional,” and “mental” exhausting levels (Pines and Aronson, 1988). Through literature review, a 10-item BMS (Burnout Measure Short Version Scale) developed by Malach-Pines (2005) has been used to measure burnout for this study. Based on these insights, this study postulated the variables of PsyCap and burnout in the perspective of entrepreneurs of Sindh, Pakistan, which is given below.

*H2*: PsyCap is negatively correlated to the Burnout of the entrepreneurs.

Given COR theory, PsyCap works as a defense resource and buffers against the mental, physical and emotional exhaustion caused by the entrepreneurial stress.

#### Burnout as a mediator

The burnout has been used as a mediating variable to explain the relationship mechanism between PsyCap and PWB. The study reported that the burnout significantly mediated the relationship between PsyCap and subjective wellbeing among the medical staff (Fan et al., 2024). Since, entrepreneurs’ task is not easy; it is full of challenges (Sheehan and St-Jean, 2014). They face stress in starting and running novel businesses (Cadet and Chasseigne, 2012). Continuous exposure to stressors causes burnout (Salami, 2011). Entrepreneurs experience burnout caused by the continuous stress of highly demanding entrepreneurial activities. Entrepreneurs’ insufficient positive psychological resources and abilities to meet those demands lead to physical and emotional reactions (Naik, 2012). For entrepreneurs, PWB is recognition of their potential and strengths which assist them in positive functioning in spending lives full of meaning, and determination, building trusting ties, dealing with uncertain and risky situations and achieving targets in entrepreneurship (Ryff and Keyes, 1995; Ryff and Singer, 2008). On the other hand, PsyCap is a “positive psychological resource” consisting of “hope, efficacy, resilience, and optimism,” and influence as a whole on the “attitudes, behaviors, performance, and wellbeing” of a person (Luthans, 2002; Luthans et al., 2004; Luthans and Youssef, 2004; Luthans and Youssef-Morgan, 2017). By engaging in entrepreneurship people fulfil their psychological desires which ultimately help them gain PWB and flourishing lives (Williams and Shepherd, 2016). Conversely, due to inadequate mental resources and abilities and increasing demands of entrepreneurial work, entrepreneurs feel burnout which badly impacts their wellbeing and health. Insights of the study witnessed that relationship between PsyCap and PWB was partially mediated in the presence of mediator burnout (Manzano-García and Ayala, 2017). Further, it is reported that PsyCap assets of employees (“self-efficacy,” “hope,” “optimism,” and “resilience”) guarded them and they experienced fewer shocks of burnout and also supported in improving PWB. This guided this study’s authors to assume that

*H3*: Burnout mediates the relationship between PsyCap and PWB.

COR theory argues that burnout depletes psychological resources and lowers PWB of entrepreneurs. However, it is proposed that PsyCap reduces burnout (resources depletion) that help entrepreneurs achieve greater PWB.

#### Conceptual model and hypothesized Links

**Figure 1:**
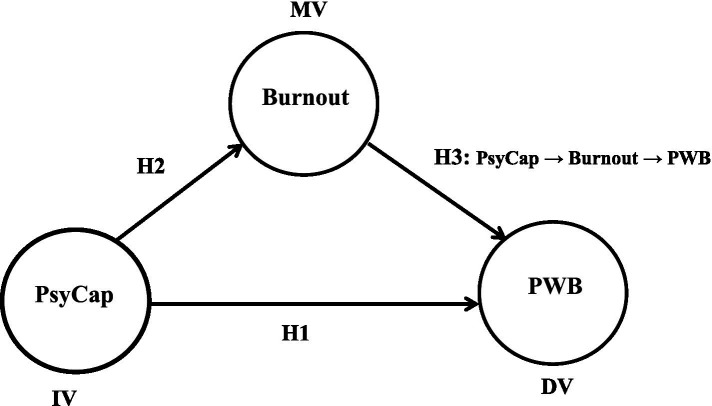
Conceptual framework of this study. Source: This study.

This study model (see Figure 1) illustrates that PsyCap is the independent variable (IV), PWB is the dependent variable (DV) and burnout is the mediating variable (MV). The hypothesized link H1 shows the direct positive correlation of PsyCap on PWB. H2 indicates the direct negative correlation of PsyCap with burnout and H3 displays the indirect relationship between PsyCap and PWB through mediator burnout.

## Research design and methodology

This empirical study employs a quantitative and cross-sectional approach to answer research questions and examine the relationships among PsyCap, Psychological wellbeing and burnout in the entrepreneurs. The simple random sampling technique has been used to give equal chance of representation to the target population (entrepreneurs). The sampling frame included the entrepreneurs who were actively engaged in operating SMEs (Small and medium enterprises) businesses for at least 1 year in three major Urban Cities of Sindh-Pakistan, such as Karachi, Hyderabad and Sukkur. The sample size determined at a confidence level of 95% in G* power is 276. The data were collected through a survey questionnaire. 309 responses were received out of 400 questionnaires which showed a response rate of 77.25%.

The data collection instrument from the past relevant studies consisted of 40 items, including 12 items of PsyCap (Avey et al., 2011), 10 items of Burnout (Malach-Pines, 2005), and 18 items of Psychological Wellbeing construct (Ryff and Keyes, 1995). Items of PsyCap and Psychological Wellbeing were rated on a Likert 7-point agreement scale (1 for strongly disagree and 7 for Strongly Agree), and for burnout items Likert 7-point frequency rating scale (1 for never and 7 for always) has been used.

## Results

The demographic data analysis in Table 1 illustrates that 90% male and 10% female respondents were starting and running businesses in different industries such as services, manufacturing, retailing and others. The table shows entrepreneurs’ age group, marital status and education level. Table 2 indicates the Mean analysis of the responses on variables based on the survey instrument rated on a Likert 7-point scale.

**Table 1:** Descriptive statistics_respondents’ profile.

Demographic characteristics	Frequency	Percent (%)
Gender		
Male	278	90.0
Female	31	10.0
Age Group		
21–30	76	24.6
31–40	155	50.2
41–50	59	19.1
Above 50	19	6.1
Marital status		
Single	94	30.4
Married	215	69.6
Education level		
Matric- intermediate	61	19.7
Graduate- masters	204	66.0
M. Phil.- PhD	44	14.2

Source: This study, *N* = 309.

**Table 2:** Mean Analysis of the respondents’ responses.

Name	No.	Mean	Standard deviation	Excess kurtosis	Skewness	Cramér-von Mises *p*-value
HOPE_1	1	5.401	1.196	−0.831	−0.232	0
HOPE_2	2	5.207	1.186	−0.416	−0.314	0
HOPE_3	3	5.57	1.109	−0.336	−0.52	0
OPTI_1	4	5.673	1.174	0.629	−0.849	0
OPTI_2	5	5.447	1.118	−0.191	−0.417	0
OPTI_3	6	5.45	1.207	0.29	−0.686	0
RESI_1	7	5.395	1.196	−0.806	−0.252	0
RESI_2	8	5.314	1.231	−0.597	−0.324	0
RESI_3	9	5.417	1.189	−0.851	−0.214	0
SE_1	10	5.392	1.185	−0.802	−0.268	0
SE_2	11	5.45	1.144	−0.519	−0.319	0
SE_3	12	5.401	1.135	−0.494	−0.301	0
SA_1	13	4.346	1.201	−0.673	0.354	0
SA_2	14	4.625	1.247	−0.671	−0.065	0
SA_3	15	3.816	1.278	0.19	0.003	0
EM_1	16	4.314	1.673	−0.742	−0.059	0
EM_2	17	4.767	1.245	−0.102	−0.409	0
EM_3	18	4.718	1.303	−0.552	−0.234	0
PIL_1	19	4.66	1.299	−0.371	−0.24	0
PIL_2	20	5.201	1.302	−0.572	−0.324	0
PIL_3	21	4.262	1.736	−1.078	−0.01	0
PG_1	22	5.835	1.242	−0.721	−0.641	0
PG_2	23	5.126	1.295	−0.518	−0.299	0
PG_3	24	4.405	1.718	−0.853	−0.185	0
PR_1	25	5.35	1.247	−0.797	−0.233	0
PR_2	26	5.129	1.203	−0.86	0.131	0
PR_3	27	5.139	1.521	0.043	−0.73	0
AUTO_1	28	4.453	1.356	−0.752	0.154	0
AUTO_2	29	4.531	1.352	−0.662	0.061	0
AUTO_3	30	4.479	1.304	−0.737	0.187	0
B_1	31	4.049	0.821	−0.742	−0.055	0
B_2	32	4.036	0.86	0.094	0.269	0
B_3	33	3.948	0.834	−0.53	−0.003	0
B_4	34	3.871	0.786	−0.368	0.153	0
B_5	35	3.99	0.919	0.175	0.22	0
B_6	36	3.861	0.79	−0.26	0.095	0
B_7	37	3.913	0.814	−0.323	0.126	0
B_8	38	3.773	0.821	0.041	0.159	0
B_9	39	3.809	0.866	0.499	−0.371	0
B_10	40	3.951	0.805	−0.583	0.201	0

Source: This study.

### PLS_SEM results

Partial Least Square-Structural Equation Modeling (PLS-SEM) has been used through Smart-PLS software version 4 by Ringle et al. (2012) to analyze quantitative data, get final results and test the hypotheses of this study. The purpose of using PLS-SEM is due to the complex structure of our model as it contains PsyCap and Psychological wellbeing as higher-order constructs with a small sample size. Furthermore, PLS-SEM is Smart-PLS making it easy to test the mediating effect of burnout in our model.

Two-stage approach “embedded two-stage approach” proposed by Ringle et al. (2012) has been used due to higher-order constructs of our model such as PsyCap and Psychological wellbeing. In the two-stage approach in the first stage the latent variable scores of lower-order components are computed, these values are then used as the manifest variables for the HOC in the second stage (Hair et al., 2018).

### Measurement model evaluation for the 1st order/lower order constructs

**Figure 2:**
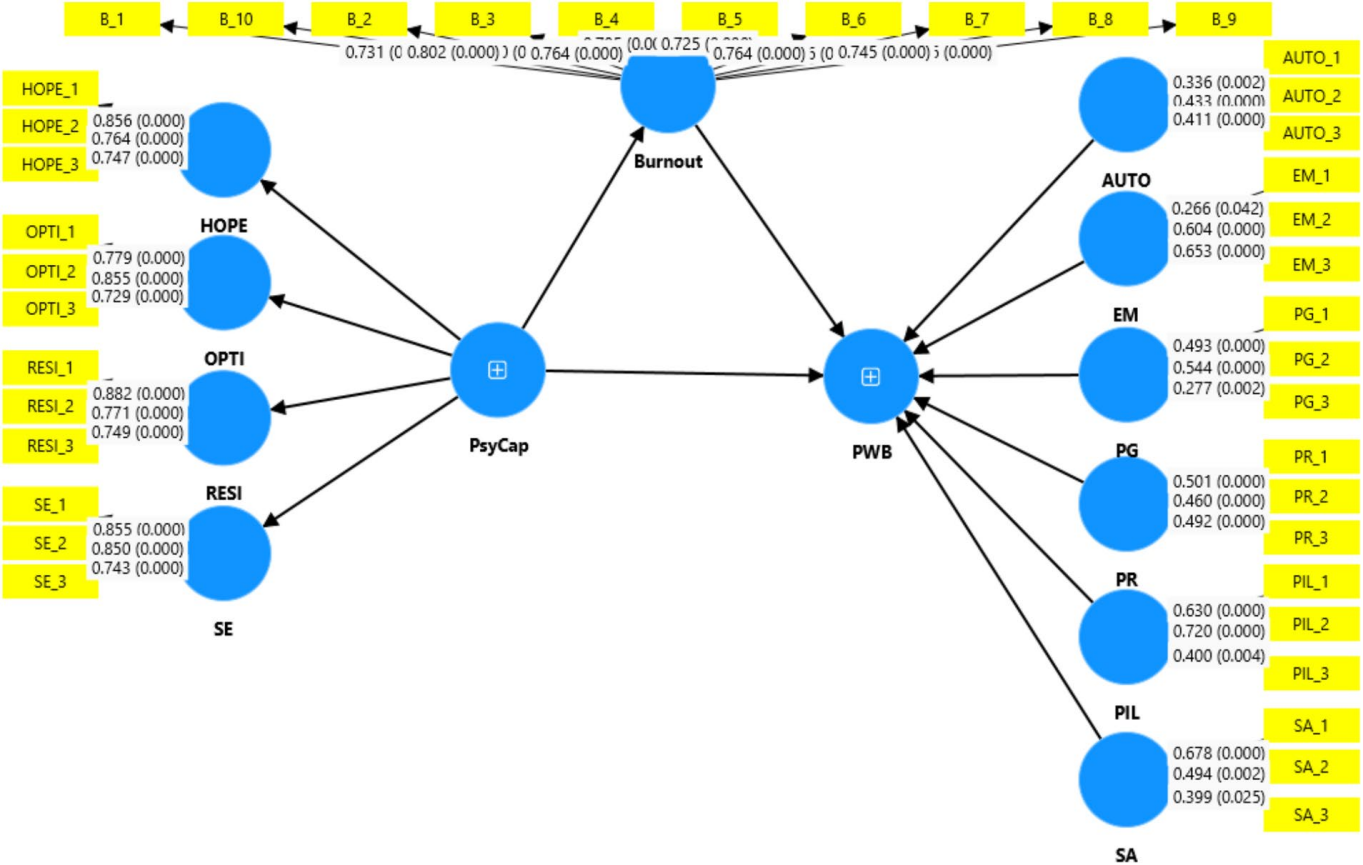
First Stage_measurement model evaluation. Source: This study.

Table 3 indicates lower-order reflective measurement model results for indicators reliability, construct reliability and convergent validity. The factor loadings values of 10 burnout items and 12 PsyCap (Hope, Optimism, resilience and self-efficacy) items are above 0.70 (see Figure 2) which confirms the reliability of outer model indicators of this study as recommended by authors (Hair et al., 2021; Hulland, 1999). In addition, Cronbach’s alpha and composite reliability values are also higher than 0.70, this suggests that constructs in this study model are reliable to measure for what they intend to measure (see Table 3). Convergent validity refers to the extent to which a construct converges and explains the variance in indicators of the construct (Hair et al., 2021; Hair et al., 2019). The convergent validity is assessed through AVE (Average Variance Extracted) and Table 3 reveals that all AVE values of constructs are above the required threshold of 0.5 which explains more than 50% required variance in indicators of the constructs.

**Table 3:** Loadings, reliability, and validity.

Items	Loadings	Cronbach’s alpha	Composite reliability (rho_a)	Composite reliability (rho_c)	Average variance extracted (AVE)
B_1	0.731	0.914	0.924	0.927	0.561
B_2	0.730				
B_3	0.764				
B_4	0.785				
B_5	0.725				
B_6	0.764				
B_7	0.726				
B_8	0.745				
B_9	0.716				
B_10	0.802				
HOPE_1	0.856	0.708	0.754	0.833	0.625
HOPE_2	0.764				
HOPE_3	0.747				
OPTI_1	0.779	0.702	0.740	0.832	0.623
OPTI_2	0.855				
OPTI_3	0.729				
RESI_1	0.882	0.724	0.756	0.844	0.645
RESI_2	0.771				
RESI_3	0.749				
SE_1	0.855	0.753	0.773	0.858	0.669
SE_2	0.850				
SE_3	0.743				

Source: This study.

Following the authors’ guidelines, this study also evaluated discriminant validity, which refers to the degree to which how much a construct varies from other constructs in the empirical sense in the model (Chin, 2010; Hair et al., 2021; Hair et al., 2019). In addition, the authors suggested using the “Fornell and Larcker Criterion” and “hetrotrait-monotrait (HTMT) ratio” to check the discriminant validity of the reflective measurement model. Table 4 about Fornell and Lacker Criterion reports that bold italic diagonal values (*0.749, 0.790, 0.789, 0.803, and 0.818*) are the squared root of AVE of the construct and are greater than correlated values of other constructs under them and hence suggest that discriminant validity has been established. HTMT ratio suggested by Henseler et al. (2015) is another way to ascertain discriminant validity and authors also recommended its value should be less than 0.85 and 0.90. Table 5 reveals that HTMT values are lower than the required threshold of 0.85 and 0.90, thus discriminant validity is created among variables of the model under study.

To assess formative lower-order indicators in the measurement model, bootstrapping was run to know the significance of outer weights. Table 6 shows that outer weights of the formative construct (Psychological wellbeing) indicators are significant (t-values > 1.98 and *p* < 0.05) and VIF are also below the required limit of 3 & 5 which shows no multicollinearity issue in our model, as suggested by authors (Hair et al., 2017).

**Table 4:** Fornell-Larcker criterion.

Constructs/dimensions	Burnout	HOPE	OPTI	RESI	SE
Burnout	***0.749***				
HOPE	−0.236	***0.790***			
OPTI	−0.142	0.648	***0.789***		
RESI	−0.133	0.749	0.642	***0.803***	
SE	−0.154	0.742	0.703	0.801	***0.818***

Source: This study. The bold italic diagonal values represent the square root of the AVE for each construct.

**Table 5:** Heterotrait-Monotrait ratio (HTMT)-matrix.

Constructs/dimensions	Burnout	HOPE	OPTI	RESI	SE
Burnout					
HOPE	0.285				
OPTI	0.164	0.885			
RESI	0.161	0.846	0.860		
SE	0.184	0.837	0.805	0.875	

Source: This study.

**Table 6:** Outer weights, significance, and VIF.

Items	Original sample (O)	Sample mean (M)	Standard deviation (STDEV)	T statistics (|O/STDEV|)	*p*-values	VIF
AUTO_1 → AUTO	0.336	0.339	0.109	3.068	0.002	3.524
AUTO_2 → AUTO	0.433	0.429	0.108	4.024	0.000	3.591
AUTO_3 → AUTO	0.411	0.407	0.075	5.462	0.000	1.291
EM_1 → EM	0.266	0.261	0.131	2.029	0.042	1.028
EM_2 → EM	0.604	0.596	0.086	7.044	0.000	1.057
EM_3 → EM	0.653	0.642	0.089	7.309	0.000	1.076
PG_1 → PG	0.493	0.485	0.086	5.750	0.000	1.577
PG_2 → PG	0.544	0.546	0.078	7.018	0.000	1.543
PG_3 → PG	0.277	0.273	0.088	3.137	0.002	1.028
PIL_1 → PIL	0.630	0.614	0.132	4.790	0.000	1.095
PIL_2 → PIL	0.720	0.702	0.097	7.458	0.000	1.010
PIL_3 → PIL	0.400	0.395	0.138	2.908	0.004	1.103
PR_1 → PR	0.501	0.489	0.102	4.915	0.000	1.188
PR_2 → PR	0.460	0.461	0.111	4.147	0.000	1.061
PR_3 → PR	0.492	0.485	0.107	4.614	0.000	1.124
SA_1 → SA	0.678	0.649	0.143	4.726	0.000	1.026
SA_2 → SA	0.494	0.478	0.163	3.032	0.002	1.030
SA_3 → SA	0.399	0.392	0.178	2.242	0.025	1.004

Source: This study.

### Measurement model evaluation for the 2nd order/higher order constructs

**Figure 3:**
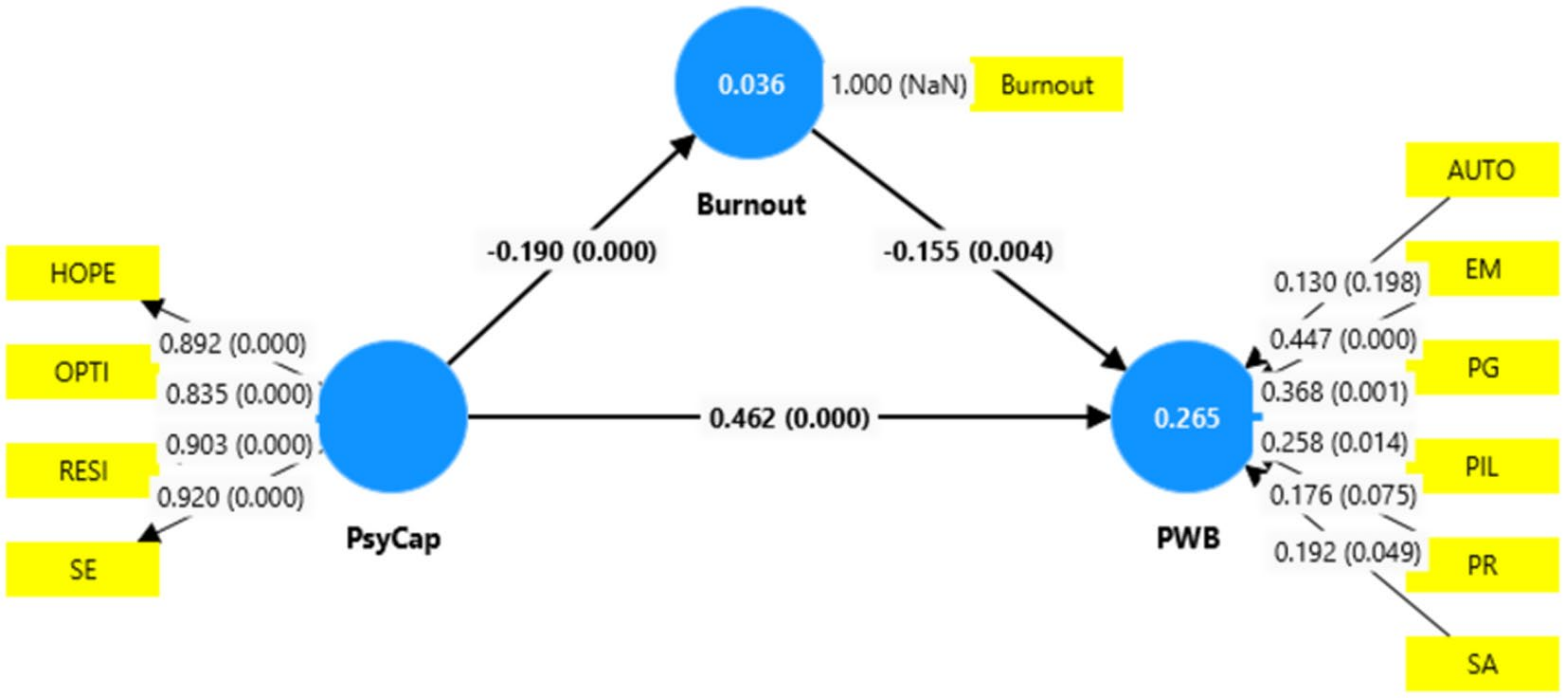
Second stage_measurement model evaluation. Source: This study.

In the second stage, the measurement model for reflective and formative indicators for higher order, created from latent values at the first stage, was evaluated similarly as in the first stage (Chin, 2010). In the reflective measurement model assessment, all indicators of PsyCap (Hope, Optimism, Resilience and Self-efficacy) have loadings greater than 0.70 (see Table 7 and Figure 3) which suggests indicators reliability as per authors’ guidelines (Hair et al., 2021; Hulland, 1999). PsyCap construct has Cronbach’s Alpha and composite reliability values above 0.70, which confirm construct reliability. In addition, AVE is also greater than 0.50 which ascertains the required convergent validity of the model (see Table 7). Tables 8, 9 indicate that discriminant validity has been established as in Fornell & Larcker Criterion the diagonal bold italic values are greater than correlated values and HTMT values are below the required threshold of 0.85 and 0.90, as recommended by authors (Hair et al., 2021; Henseler et al., 2015).

**Table 7:** Loadings, reliability, and validity.

Items	Loadings	Cronbach’s alpha	Composite reliability (rho_a)	Composite reliability (rho_c)	Average variance extracted (AVE)
Burnout	1				
HOPE	0.893	0.911	0.915	0.937	0.789
OPTI	0.836				
RESI	0.903				
SE	0.920				

Source: This study.

**Table 8:** Discriminant Validity_ Fornell-Larcker criterion.

Constructs	Burnout	PsyCap
Burnout	***1***	
PsyCap	−0.190	***0.888***

Source: This study. The bold italic diagonal values represent the square root of the AVE for each construct.

**Table 9:** Discriminant validity_Heterotrait-Monotrait ratio (HTMT)-matrix.

Constructs	Burnout	PsyCap
Burnout		
PsyCap	0.196	

Source: This study.

Formative measurement model for higher-order construct Psychological wellbeing was assessed by computing weights significance or loadings significance and VIF values through bootstrapping procedure in PLS-SEM. Table 10 reports that Psychological wellbeing indicators such as EM, PG, PIL and SA have significant outer weights but two indicators AUTO and PR have non-significant outer weights, however, their outer loadings were significant so they were retained in the model. In addition, VIF values are below 0.3, which suggests no collinearity issues in the model.

**Table 10:** Outer weights significance/loadings significance and VIF.

Items/constructs	Weights, *t*-values and *P*-values			Loadings with *P*-values	VIF
AUTO → PWB	0.130	1.289	0.198	0.534 (0.000)	1.272
EM → PWB	0.447	4.370	0.000	0.734 (0.000)	1.223
PG → PWB	0.368	3.379	0.001	0.712 (0.000)	1.331
PIL → PWB	0.258	2.447	0.014	0.616 (0.000)	1.219
PR → PWB	0.176	1.783	0.075	0.514 (0.000)	1.209
SA → PWB	0.192	1.973	0.049	0.475 (0.000)	1.114

Source: This study.

### Structural path model evaluation and hypotheses testing

The structural Path model which is also referred to as the inner model is assessed for Path coefficients significance, collinearity issues, model’s explanatory power (R^2^), predictive relevance (Q^2^ predict) and effect size (f ^2^) as recommended by authors (Hair et al., 2021).

**Figure 4:**
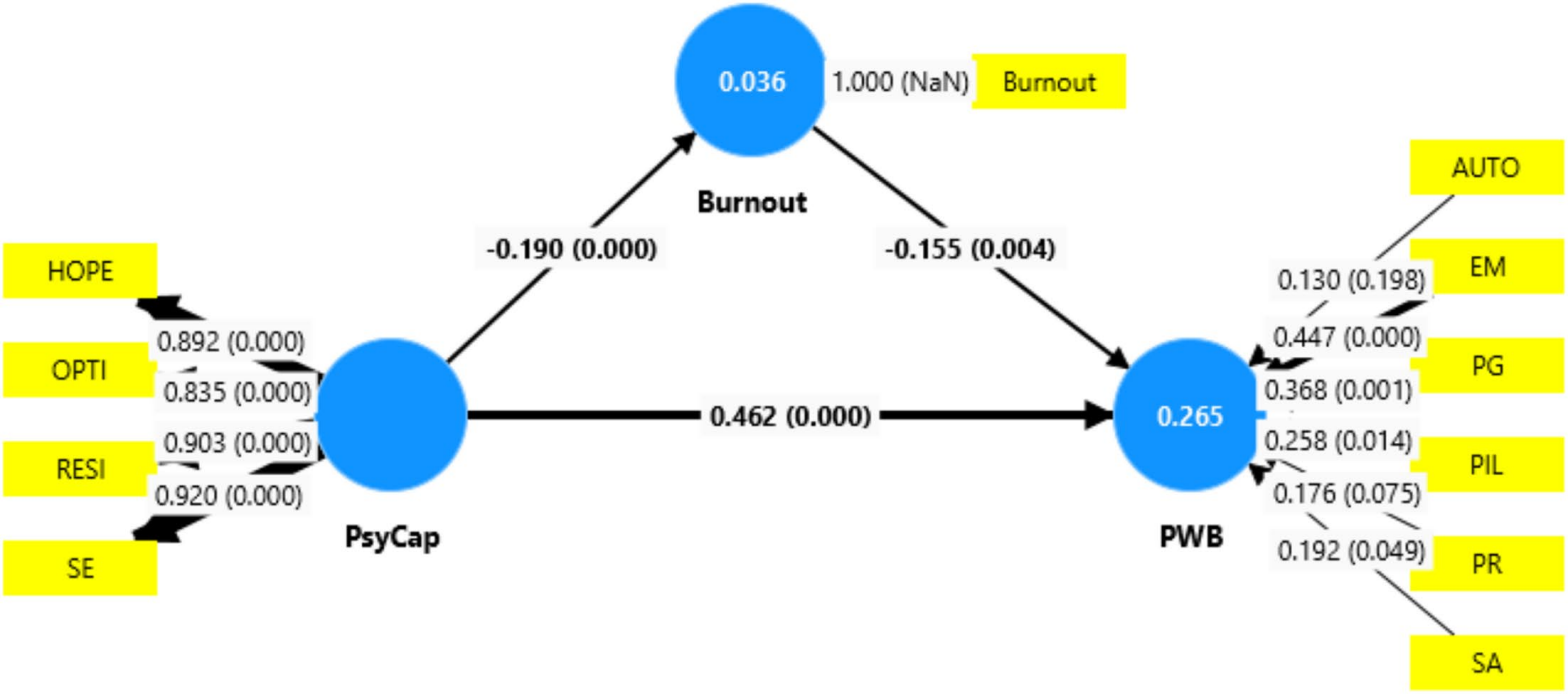
Final path model_structural model assessment. Source: This study.

The bootstrapping procedure was run in PLS-SEM to get structural model evaluation results. Table 11 shows (see Figure 4) that PsyCap is positively correlated to PWB in a significant way (PsyCap → PWB, *β* = 0.462, *t* = 10. 301, and *p* < 0.05). The results further report that PsyCap is negatively correlated to burnout which is also significant (PsyCap → Burnout, *β* = −0.190, *t* = 3.176, and *p* < 0.05). Furthermore, the relationship of Burnout with PWB is also negative and significant (Burnout → PWB, β = −0.155, *t =* 2.846, and *p <* 0.05). The significance of path coefficients was also assessed with the bootstrapping method through suggested confidence interval bias-corrected (Aguirre-Urreta and Rönkkö, 2018). It can be seen in Table 11 that at a limit of 5% probability, zero does not fall into 95% (confidence interval bias-corrected) among upper and lower bounds. This study’s structural model has no collinearity issues as VIF are below the recommended threshold of 3 (see Table 11).

**Table 11:** Path coefficients (mean, STDEV, *t-*values, and *p*-values).

Paths	β	(STDEV)	T statistics	*P*-values	2.5%	97.5%	VIF
PsyCap → PWB	0.462	0.045	10.301	0.000	0.353	0.535	1.038
PsyCap → Burnout	−0.190	0.051	3.716	0.000	−0.289	−0.086	1.000
Burnout → PWB	−0.155	0.054	2.846	0.004	−0.254	−0.039	1.038

Source: This study.

This stuy model’s explanatory power is assessed through coefficient of determination *(R^2^)* values, which are shown in Table 12. The table further indicates the Q^2^ predict values are greater than zero, which confirms the required threshold; it is also referred to as predictive relevance and reflects the model’s predictive accuracy. In addition, f-square, the effect sizes are also reported (see Table 12).

**Table 12:** R^2^, Q^2^ predict and f ^2^.

LV	R-square	Q^2^ predict
Burnout	0.036	0.030
PWB	0.265	0.214
Paths	f-square	
Burnout → PWB	0.034	
PsyCap → Burnout	0.038	
PsyCap → PWB	0.276	

Source: This study.

### Mediation analysis (burnout as a mediator)

The indirect effect, through burnout mediator, was assessed in bootstrapping procedure. Results in Table 13 reveal that the total effect of PsyCap on PWB is (*β* = 0.492), which is greater than the direct effect (β = 0.46). This guides that there is mediation of burnout and indirect effect passes from PsyCap to PWB through burnout mediator (PsyCap → Burnout → PWB, β = 0.029) which is significant too (*t* = 2.144, *p* < 0.05).

**Table 13:** Indirect effect (mediation analysis).

Effect type	Paths	β	SD	*t*-values	*P*-values
Total effect	PsyCap → PWB	0.492	0.042	11.834	0.000
Direct effect	PsyCap → PWB	0.462	0.045	10.301	0.000
Indirect effect	PsyCap → Burnout → PWB	0.029	0.014	2.144	0.032

Source: This study.

## Discussion

The results of this study in the context of hypothesized relationships among variables PsyCap, PWB and burnout from the perspective of entrepreneurs of Sindh, Pakistan are discussed here. These also debate the theoretical validation which is supported with prior empirical evidence.

*H1*: PsyCap is positively correlated to the PWB of the entrepreneurs.

The hypotheses testing results (see Table 11) show that PsyCap is positively correlated to the PWB of entrepreneurs (PsyCap → PWB, β = 0.462, *t* = 10. 301, and *p* < 0.05) which confirms hypothesis (H1) of the study. This significant relationship guides that entrepreneurs with strong PsyCap resources such as hope, optimism, resilience and self-efficacy can have good PWB during their entrepreneurship activities; similar findings were also reported by authors (Al Kahtani and M, 2022; Culbertson et al., 2010; Hansen et al., 2015; Hmieleski and Carr, 2007; Semmer et al., 2008). While entrepreneurs in Sindh (Pakistan) face heavy stressors caused by economic, social and environmental challenges, which burnout and subsequently worsen PWB. Therefore, PsyCap resources become vital for entrepreneurs of Sindh to cope with burnout and achieve better PWB. This is also consistent with empirical evidence from previous studies (Baluku et al., 2018; Malekitabar et al., 2017). In other words, it can be said that positive psychological resources such as “hope,” “optimism,” “resilience” and “self-efficacy” keep entrepreneurs strong, and hopeful about achieving goals and make them resilient to face and handle risks and unexpected situations during entrepreneurial activities. These positive aspects keep entrepreneurs motivated and engaged in business and as a result, they achieve greater wellbeing by satisfying psychological needs (Shir et al., 2019). The findings of this study also validate Hobfoll (1988, 1989) “conservation of resources (COR) theory” which talks about the “resources gain” and “resources loss.” This theoretical base supports our research outcomes which argue that entrepreneurs of Sindh (Pakistan) due to uncertainty and risks involved in businesses, economic and environmental challenges and other problems experience stress cause the depletion (loss) of personal psychological resources, social resources and material resources. This reduction of entrepreneurial resources leads to burnout which subsequently lowers the PWB of entrepreneurs; however, resources gain which is replenishing personal resources that increase PsyCap factors (hope, optimism, resilience and self-efficacy) would buffer the negative effects of burnout and improve the PWB of entrepreneurs. Entrepreneurs fulfil psychological needs by involving in business and entrepreneurship. Therefore, conservation of psychological capital resources is key to entrepreneurs to use them in case of loss of resources. This reservoir of psychological assets such as hope, optimism, resilience and self-efficacy would help entrepreneurs to refill and utilize in gratifying psychological needs in current and also use in coping with uncertainties and challenges of future. Through this, the entrepreneurs in Sindh would achieve good PWB, enjoy their flourishing, meaningful and fulfilling lives and contribute efforts to the economic growth of Sindh and Pakistan.

H2: PsyCap is negatively correlated to the Burnout of the entrepreneurs.

In the case of the PsyCap link with burnout in our study model, the findings (see Table 11) reveal that PsyCap negatively correlates to burnout (PsyCap → Burnout, *β* = −0.190, *t* = 3.176, and *p* < 0.05) which confirms hypothesis (H2) of this study. This means that entrepreneurs with greater levels of PsyCap support them in reducing the worst consequences of burnout, prior evidence also supports this (Baron et al., 2016; Hmieleski and Carr, 2007; Malekitabar et al., 2017). Entrepreneurship is a more demanding job which involves risks and uncertainties and entrepreneurs due to limited abilities and PsyCap resources feel stress and burnout (Demerouti et al., 2001). This is also true for the entrepreneurs of Sindh (Pakistan). Therefore, the entrepreneurs of Sindh require adequate resources of hope, optimism, resilience and self-efficacy which reduce burnout produced by economic and environmental uncertainties and more demanding tasks. In this context, researchers Malak et al. (2022) also found that PsyCap reduces burnout and helps entrepreneurs gain success in their business activities. In another way, it can be said that nurturing and upholding PsyCap resources would assist entrepreneurs to remain resilient and cope with burnout and stressful situations (Fredrickson, 1998). With this, the entrepreneurs in Sindh can achieve entrepreneurial goals by ameliorating the bad effects of burnout through PsyCap assets.

*H3*: Burnout mediates the relationship between PsyCap and PWB.

Burnout is the mediating variable in our study model (see Figure 4). It is observed in the results in Table 13 that there is an indirect and significant relationship between PsyCap and PWB through mediator burnout (PsyCap → Burnout → PWB, β = 0.029, *t* = 2.144, *p* < 0.05). This confirms hypothesis (H3) and is also consistent with the results of the authors’ research (Manzano-García and Ayala, 2017). Findings of previous studies maintained that people engage in entrepreneurship activities to satisfy their psychological needs and this helps them to achieve better PWB (Shepherd and Patzelt, 2017; Williams and Shepherd, 2016), thus, this also mitigates the negative effects of burnout that emerges from entrepreneurship work (Abreu et al., 2019; Shir et al., 2019). Entrepreneurship requires great efforts and mental resources. In Pakistan, entrepreneurs face more challenges. This demands entrepreneurs to put more energy on activities for starting, growing and developing businesses, which cause burnout (Demerouti et al., 2001). Higher risks and uncertainties cause entrepreneurs to face more burnout (Harris et al., 1999; Monsen and Wayne Boss, 2009). Although results report that burnout hurts the PWB of entrepreneurs and hampers their optimum Psychological functioning to achieve their thriving and purposeful lives, yet, the healthier PsyCap assets including “optimism,” “resilience,” “hope” and “self-efficacy” enable entrepreneurs to safeguard from burnout and fulfil their psychological desires and improve PWB (Baluku et al., 2018; Hmieleski and Carr, 2007). Entrepreneurs’ loss of psychological resources due to burnout are replenished through improved PsyCap. This helps entrepreneurs to be resilient and cope with undesirable situations of current and upcoming times (Hobfoll et al., 2018).

### Implications of the study

Theoretically, this study confirms the COR theory in the context of entrepreneurship. Moreover, it provides the theory-based framework for understanding the mechanism of psychological resources loss and resources gain under the stressors, which lead to burnout. Practically, the current study fills the research gap by providing empirical evidence on PsyCap, PWB and burnout and guides the significance of PsyCap resources for entrepreneurs’ positive psychological functioning and wellbeing. The findings of this study contribute to academic institutions and higher education authorities in making policies for developing PsyCap resources and PWB of young potential entrepreneurs. Additionally, the research outcomes provide the fresh insights about the mediating role of burnout on the relationship between PsyCap and PWB of entrepreneurs.

### Limitations

The study has limitations as the data were collected from the entrepreneurs of the urban cities of the Sindh region of Pakistan. However, the research findings can be generalized in context of the urban population as the study addresses the research question and research gap. Though this research is limited in using quantitative methods, however, qualitative and mixed methods can also be applied to get an in-depth understanding.

### Future research directions

The authors of this study suggest that future research can contribute insights on same variables in rural settings. Investigations can also use gender as a moderating variable to examine the variations of results among male and female entrepreneurs. Upcoming research can also focus on using PsyCap with other variables such as emotional intelligence, sustainability, diversity and work-life balance in the context of entrepreneurs. Furthermore, along with PsyCap and PWB other variables such as entrepreneurs’ socioeconomic status, access to financing and support networks provide a gap for investigation in cultural context. In addition, future researchers can explore the dark side of psychological wellbeing in the entrepreneurs. New researches can also focus on stress and burnout in the digital and AI age and maintaining mental health and wellbeing issues.

## Conclusion

This study concludes that PsyCap is a very important psychological resource which is obligatory for entrepreneurs to perform entrepreneurial activities and maintain better PWB. PsyCap also supports entrepreneurs in situations which involve more risks and challenges through coping and resilience. Burnout disturbs the positive psychological functioning of entrepreneurs and reduces PWB, however, the healthier PsyCap resources buffer the negative effects of burnout and facilitate entrepreneurs to fulfil psychological desires and achieve thriving and purposeful lives. Thus, in developing countries like Pakistan, inappropriate facilities, increasing economic challenges and more risk in starting and running a business create stress for entrepreneurs. Under such stressors, if entrepreneurs lack positive psychological aspects such as hope, optimism, resilience and efficacy, then it would lead to experience burnout. The increasing levels of burnout deteriorate the PWB of entrepreneurs. Therefore, as research findings revealed PsyCap resources reduce burnout and enhance PWB. This suggests that entrepreneurs need to grow ample assets of PsyCap for coping worst situations in businesses and achieving better PWB. The present study answers the research question on burnout as a mediating variable and supplies valuable practical outcomes to the current literature stream. This investigation also contributes new insights into the literature and suggests new avenues for future researchers.

## Data Availability

The raw data supporting the conclusions of this article will be made available by the authors, without undue reservation.
